# In the eye of the storm: the mental health situation of PhD candidates

**DOI:** 10.1007/s40037-020-00639-4

**Published:** 2020-12-15

**Authors:** Lucille M. S. Mattijssen, Josephine E. Bergmans, Inge C. M. van der Weijden, J. Christine Teelken

**Affiliations:** 1 Department of Sociology, Vrije Universiteit Amsterdam, Amsterdam, The Netherlands; 2 Center for Science and Technology Studies, Leiden University, Leiden, The Netherlands; 3 Department of Organization Sciences, Vrije Universiteit Amsterdam, Amsterdam, The Netherlands

Completing a PhD is internationally considered the highest level of education that individuals can attain. It is a challenging process, that is known to be accompanied by high levels of stress, pressure and also loneliness. Despite this, until a couple of years ago, evidence about the mental health issues of PhD candidates often remained limited to anecdotal evidence. In recent years, more and more research is being carried out concerning the wellbeing of PhD candidates, as in this issue by Kusurkar et al. [1]. Going from anecdotal evidence, various studies have structurally collected both quantitative and qualitative data concerning PhD wellbeing. These studies have helped to falsify the past “low-stress” image of universities, and in contrast confirmed that the wellbeing of PhD candidates and early career researchers is of serious concern. Research by Levecque et al. [2] shows that 32% of PhD candidates in Belgium are at risk of developing mental health problems related to stress. The PhD Candidate Network Netherlands (PNN) conducted a survey amongst 1600 PhD candidates between March and May 2020, a period during a so-called intelligent lockdown to prevent the spread of COVID-19, which meant that more sectors were open than in other countries with stricter lockdown. This survey showed that no less than 47% of PhD candidates in the Netherlands were at risk of developing a psychiatric disorder, 39% showed severe symptoms of burnout and 40% experienced a high or very high workload [3]. Research published in *Nature* in 2019 [4] regarding 6300 PhD candidates from all over the world found that 36% of PhD candidates had sought help for anxiety or depression caused by their PhD trajectory. Combined, these studies confirm that mental health problems are widespread during the PhD trajectory, and not limited to specific countries or contexts.

These studies also identify factors that influence the mental wellbeing of PhD candidates. In a comprehensive overview of 163 studies on PhD candidate wellbeing, Sverdlik et al. [5] identified four main external factors (supervision, personal life, departmental structures and financial opportunities) and five main internal factors (motivation, writing skills, academic identity, self-worth and self-efficacy) that influence PhD wellbeing. The PNN survey [3] furthermore found international PhD candidates to be more at risk of mental health problems compared to their domestic colleagues. Some studies look outside the workplace for potential factors that may negatively impact the mental wellbeing of PhDs. Van der Weijden and Bergmans [6], for instance, showed that PhD candidates in the Netherlands who give informal care (almost 30%) to a loved one develop feelings of constant strain, inability to overcome difficulties, and sleeping problems. The combination of informal care responsibilities, doing a doctoral study and having private and family life at the same time could result in mental health problems, as 55% of informal caregiving PhD candidates were at risk of developing a psychiatric disorder.

This study by Kusurkar et al. [1] complements these existing findings in two ways. First of all, most other research focuses mainly on stressors and risks factors. While one could say that the opposite of stressors are mitigators (e.g. get rid of bad work-family balance, have a good supervisor rather than a bad one, stop being unmotivated), Kusurkar et al. [1] start from the positive side and ask PhD candidates explicitly about moderating factors and factors that energize their PhD study. In this way, the focus lies more on what PhD candidates should do rather than what they should not. Experiencing affiliation with colleagues, focusing on concrete achievements and intrinsic motivation are the most frequently listed energizers. Secondly, the researchers investigate the interplay between various factors, exposing the mechanisms via which both stressors and energizers affect the various dimensions of burnout and how these are interrelated.

Due to these studies, PhD candidate wellbeing is getting more and more attention from stakeholders and policy makers. More and more universities and medical centers have implemented policies aiming to improve the mental wellbeing of PhD candidates and early career researchers, offering various forms of support and training. However, attention to PhD wellbeing is not limited to local institutions. At the international level, the OECD Global Science Forum (2019–2020) is now conducting a project that aims to reduce the precarity of research careers. The main purposes are to identify policies and procedures that could support better strategic planning and management of research careers, while increasing the wellbeing of researchers and the quality of their research. Though this project focuses mostly on postdocs, improvements resulting from this project are likely to be helpful for PhD candidates as well.

These recent efforts to improve the mental wellbeing of PhD candidates have gained even more importance since the outbreak of COVID-19 and the measures aimed to prevent further spread of the virus. Candidates experience delays in their projects due to the closing down of research facilities, difficulties in pursuing data collection, an inability to do fieldwork or a struggle to work from home due to care duties, suboptimal home working conditions or worries about the pandemic [3]. In these times, this research field offers institutions insights into how they can help their PhD candidates and early career researchers to get through these difficult times. However, as these times are unprecedented, additional research is required to identify whether the pandemic has impacted the wellbeing of PhD candidates, either positively or negatively, and which factors can help to (re)generate resilience, ensuring that they can successfully and healthily complete their trajectories.
